# Pistol Shooting Dystonia Treated with Thalamotomy

**DOI:** 10.5334/tohm.779

**Published:** 2023-09-04

**Authors:** Masahiko Nishitani, Shiro Horisawa, Taku Nonaka, Kotaro Kohara, Tatsuki Mochizuki, Takakzu Kawamata, Takaomi Taira

**Affiliations:** 1 Department of Neurosurgery, Tokyo Women’s Medical University, Tokyo, JP

**Keywords:** dystonia, thalamotomy, pistol shooting

## Abstract

**Background:**

Neurosurgical treatment for pistol shooting dystonia has not been studied.

**Case report:**

The patient was a 41-year-old woman who participated in the Olympic Games four times as a shooting player. Five months after the final Tokyo Olympic trials, she developed dystonia of the right index finger when shooting. Stereotactic thalamotomy was performed, and a complete resolution of dystonia was achieved. She garnered her personal best score and placed fifth in the Tokyo Olympics.

**Discussion:**

Thalamotomy along with deep brain stimulation can be a surgical modality for patients with task-specific dystonia who fail oral medications or botulinum toxin therapy.

## Introduction

Task-specific dystonia is a movement disorder, characterized by involuntary movement when performing a specific task [1]. It is caused by long-term repetitive and stereotypical movements [2] and a common type of this disease is the musician’s task-specific dystonia, which presents as involuntary movements when playing musical instruments [3]. Professional athletes have also developed task-specific dystonia, including but not limited to pistol-shooting, has been reported [4]. Among professional musicians, the prevalence of task-specific dystonia is approximately 1–3% [5,6]. This suggested that task-specific dystonia was common in specific populations. The initial treatment options for task-specific dystonia are oral medications, like trihexyphenidyl, clonazepam, and baclofen, as well as botulinum toxin injections [7]. Neurosurgical treatment may be considered in cases that do not respond completely to initial treatment. However, there have been few reports on the neurosurgical treatment options for task-specific dystonia among athletes. Task-specific dystonia, involving musicians and athletes, has been treated by creating ablative lesions on the ventro-oral (vo) nucleus of the thalamus (vo-thalamotomy) [8,9]. However, the degree of improvement with vo-thalamotomy varied between individual patients.

The Olympic games are the leading international sporting events, featuring numerous top-ranking athletes from more than 200 countries and regions. This report will elaborate on a case of task-specific dystonia, related to pistol-shooting (trap-shooting), that was successfully treated via vo-thalamotomy, resulting in participation in the Tokyo Olympics 2020.

## Case Report

Trap-shooting, which involves shooting at clay targets with a shotgun, was introduced to the Olympic program in 1900. The goal of trap-shooting is to hit clay targets, flying away from the shooter. The targets were shot in the air at varying angles. Trap shooters shoot five shots from five different points on a semi-circular field. Therefore, shooters fire a total of 25 shots per round. In the Olympic version of trap-shooting, a team of one male and one female receives 150 shots, with each individual having 75 shots.

**Figure 1:**
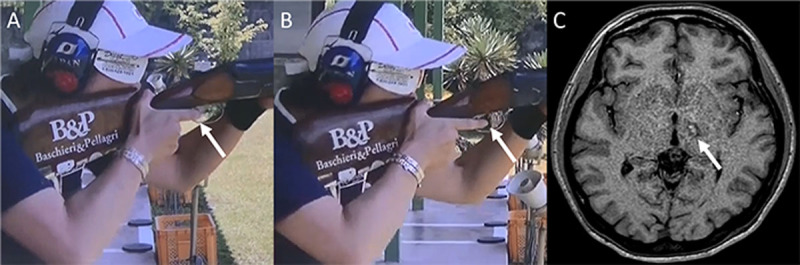
Moment of triggering and postoperative MRI. Distal interphalangeal joint of the triggering finger (arrow) flexed at pre-symptomatic condition **(A).** 2 months after the onset of dystonia, distal interphalangeal joint of the triggering finger remained extended due to dystonia at the moment of triggering **(B).** T1-weighted head MRI on the day of the surgery showing coagulated lesion (arrow) on the left thalamus **(C)**.

The patient was a 41-year-old right-handed Japanese woman, who participated in the Olympic Games four times as a shooting player and garnered a place in the Tokyo Olympics. Her medical and family histories were unremarkable. She started participating in competitive shooting at the age of 18. She participated as a shooter in the Sydney, Beijing, London, and Rio de Janeiro Olympics. Her hit ratio just before the final Tokyo Olympic trials was 91.2%. Five months after the final Tokyo Olympic trials, she noticed occasional stiffness of the right index finger (trigger finger) when shooting. Within two months after the onset of right index finger stiffness, the severity and frequency of the stiffness rapidly progressed, resulting in near-impossible depression of the trigger (Figure 1AB and Video 1). Her trap-shooting score gradually worsened, and she was unable to depress the trigger because of severe right index finger stiffness. Her hit ratio decreased to 57.6% five months after the final Tokyo Olympic trial. The involuntary stiffness of the right index finger manifested only when the patient was shooting. She had no history of trauma or dopamine receptor blocking agents. Her cranial magnetic resonance imaging was unremarkable. The probable diagnosis was task-specific focal hand dystonia. Her Burke-Fahn-Marsden Dystonia Rating Scale (BFMDRS)-right arm was 4. She rejected oral medications and botulinum toxin injections because she was highly sensitive to the doping inspection. In addition, botulinum toxin treatment was not covered by insurance for upper extremity dystonia in Japan and was not an option as a treatment. The patient strongly requested to undergo neurosurgical treatment.

**Video 1:** **Pre- and post-operative pistol shooting.** Video showing the normal and dystonic triggering before the surgery and the condition 10 months after the surgery.

Four months after the onset of her symptoms, the patient underwent stereotactic radiofrequency thermal ablation of the left VO-thalamotomy for focal hand dystonia (Figure 1C). The details of the surgical procedures have been described in our previous articles [10]. Postoperatively, no stiffness of the right index finger was observed during shooting. The postoperative BFMDRS-right arm was 0. However, the patient had mild dysarthria and decreased muscle tone on the right side of the body, resulting in reduced writing and speech, and unsteady gait. Three months postoperatively, she successfully returned to trap-shooting. Although she was able to depress the trigger without stiffness, withstanding the impact of the shot was difficult due to the decreased muscle tone on the right side of the body. Thus, she gained poorer scores seven months postoperatively (hitting ratio: 68.8% to 75.2%). After 10 months of intensive physical training, which focused on compensating for alterations in body balance and decreased muscle tone on the right side of the body, she successfully achieved her scores prior to developing dystonia (hitting ratio: 88.8%, Video 1).

During the Tokyo Olympics, which happened one year postoperatively, her hitting ratio was 92% for the female trap-shooting event, and she ranked 19^th^ out of 26 participants. During the mixed team’s event, her hitting ratio was 98.7% (her personal best for the Olympics), and she ranked fourth among 16 countries. After participating in the Tokyo Olympics, she ended her professional career with satisfaction. Currently, the patient still has mild dysarthria and decreased muscle tone on the right side of the body. The progression of her hit ratios over time is shown in Table 1.

**Table 1:** Time course of hitting ratio at trap shooting.


DATE	EVENT	HITTING RATIO	RANK

2008	Beijing Olympic Women	86%	4th/20

2012	London Olympic Women	86.70%	15th/22

2016	Rio de Janeiro Olympic Women	81.30%	20th/21

2019.11	Final Tokyo Olympic Trials (14th Asian Shooting Championship Doha)	91.20%	3th/32

2020.3	Disease Onset	79.20%	

2020.4	Practice	57.60%	

2020.5	Practice	Unable to play	

2020.7	Surgery		

2021.1	Practice	76%	

2021.2	Practice	68.80%	

2021.5	Practice	88.80%	

2021.7	Tokyo Olympic Women	92%	19th/26

	Tokyo Olympic Mixed	98.70%	4th/16


## Discussion

This is the first report of task-specific dystonia, related to pistol-shooting, in an Olympic athlete, who successfully recovered with stereotactic thalamotomy.

Prolonged stereotypical movements can induce dystonia in the involved areas [2]. This has been reported in musicians and athletes, including table tennis players, golfers, and runners. There have been two reported cases of dystonia related to pistol-shooting [4,11]. The first case presented as the inability to flex the index finger while shooting, but the treatment was not discussed [11]. The second case presented with forearm stiffness and hand-twisting when the patient, a competitive pistol-shooter, held a gun [4]. This patient received botulinum toxin injections in the affected muscles, including the extensor carpi radialis, pronator teres, flexor carpi radialis, and flexor carpi ulnaris. However, the treatment results were not documented. In the present case, the patient recovered to a pre-symptomatic level, and she achieved her personal best in the Olympics. Long-term intensive physical training was required to compensate for alterations in the muscle tone and body balance, which did not completely recover after one year postoperatively. Thalamotomy-induced complications include dysarthria, dysesthesia, and weaknessx [10,12]. These alterations in physical function result in impaired performance.

Dystonia in athletes may be misdiagnosed with a psychological origin, such as performance anxiety [13]. There is no diagnostic test that distinguishes dystonia from a psychological origin. However, task-specific dystonia is mainly characterized by dystonia, which occurs only when performing a specific task, regardless of the situation (practice or competition). The initial treatment of task-specific dystonia includes oral medications and botulinum toxin injections [7].

Stereotactic thalamotomy is indicated for dystonic patients who do not recover after initial treatment. Focused ultrasound thalamotomy, which induces thermal lesions in the deep-seated structures, without a skin incision, is also a viable treatment for task-specific dystonia [14]. However, it harbors the same complications as radiofrequency thalamotomy. Likewise, deep brain stimulation (DBS) is also an option [15]. Although DBS involves the implantation of mechanical devices, including intracranial electrodes and pulse generators, irreversible complications, such as those seen in the present case, are less likely to occur. Moreover, it provides the same degree of improvement as thalamotomy. In this patient, implantation of the instrument in the body was unacceptable. In addition, focused ultrasound was not an option because it was not covered by insurance for dystonia in Japan. Therefore, radiofrequency thermocoagulation was the treatment of choice.

GPI is most commonly used for surgery for dystonia. GPI surgery has been used for generalized dystonia and cervical dystonia. Iacono et al. reported that thalamotomy is effective for distal limb dystonia and pallidotomy is effective for proximal dystonia [16]. Tasker et al. also reported that thalamotomy is less effective for proximal dystonia [17]. Against this background, pallidotomy has not been used for focal hand dystonia. Fukaya et al. examined symptom improvement in patients with writer’s cramp by implanting electrodes on the Vo-Vim and GPi, and found that stimulation of the GPi produced little improvement in writer’s cramp, while stimulation of the Vo-Vim produced significant symptom improvement [18]. Therefore, DBS was used to target the Vo-Vim nucleus. The efficacy of thalamic Vo nucleus thalamotomy with FUS has also been reported [14]. The overwhelming majority of previous reports of surgery for focal hand dystonia have used the thalamic Vo nucleus [19,20,21,22]. Recent study reported the efficacy of Vim-DBS for writer’s cramp [23]. It remains unclear whether the Vo or Vim nucleus should be targeted for focal hand dystonia. Based on our experience, we believe that the thalamic posterior part of Vo (Vop) nucleus is an effective site for focal hand dystonia. Therefore, in order to accurately coagulate the entire thalamic Vop nucleus, we perform surgery to ablate the nucleus Vop in front of and behind the nucleus Vop. Therefore, our procedure is always accompanied by ablation of the anterior part of the thalamic Vim nucleus. Because the Vop and Vim nuclei are adjacent to each other, it is difficult to stimulate or ablate them independently. Hirt et al. reported that stimulation of the proximal contact, which is closest to the Vop nucleus, is effective for focal hand dystonia treated by Vim-DBS [23]. The stimulation of the proximal contact of Vim-DBS electrode may costimulate the Vop nucleus.

Several professional athletes have lost their careers due to incorrectly diagnosed or treated dystonia. Thus, accurate diagnosis and appropriate treatment are necessary to allow professional athletes to continue competing.
